# Consanguinity and Congenital Heart Disease Susceptibility: Insights into Rare Genetic Variations in Saudi Arabia

**DOI:** 10.3390/genes13020354

**Published:** 2022-02-16

**Authors:** Nour Albesher, Salam Massadeh, Sabah M. Hassan, Manal Alaamery

**Affiliations:** 1 KACST-BWH Centre of Excellence for Biomedicine, Joint Centers of Excellence Program, King Abdulaziz City for Science and Technology (KACST), Riyadh 12354, Saudi Arabia; nalbesher@kacst.edu.sa (N.A.); massadehsa@ngha.med.sa (S.M.); 2 Department of Biological Sciences, Faculty of Sciences, King Abdulaziz University, Jeddah 21589, Saudi Arabia; smmhasan1@kau.edu.sa; 3 Developmental Medicine Department, King Abdullah International Medical Research Center, King Saud Bin Abdulaziz University for Health Sciences, King Abdulaziz Medical City, Ministry of National Guard-Health Affairs (MNG-HA), Riyadh 11481, Saudi Arabia; 4 Saudi Human Genome Project, King Abdulaziz City for Science and Technology, Riyadh 12354, Saudi Arabia; 5 Princess Najla Bent Saud Al-Saud Center for Excellence Research in Biotechnology, King Abdulaziz University, Jeddah 21589, Saudi Arabia; 6 Department of Genetics, Faculty of Agriculture, Ain Shams University, Cairo 11241, Egypt

**Keywords:** congenital heart disease, consanguinity, Saudi Arabia, autosomal recessive, autosomal dominant

## Abstract

Congenital heart disease (CHD) encompasses a wide range of structural defects of the heart and, in many cases, the factors that predispose an individual to disease are not well understood, highlighting the remarkable complexity of CHD etiology. Evidence of familial aggregation of CHD has been demonstrated in different communities and for different cardiac lesions. Consanguinity, particularly among first cousins, is an added risk factor for these families, particularly in societies where it is considered a common cultural practice, as confirmed in previous studies conducted in Saudi Arabia and other countries. Through comprehensive genetic testing of affected families, we have been able to better understand the genetic basis of the various cardiac lesions and to delineate the molecular mechanisms involved in cardiac morphogenesis. In this review, we discuss the epidemiology and genetics of CHD in consanguineous populations focusing on Saudi Arabia as an extensive study model to address current advances and challenges in the clinical genetic diagnosis and prevention of CHD.

## 1. Introduction

Despite rising awareness, congenital heart disease (CHD) is still a major cause of significant morbidity and mortality. It is clinically defined as a group of structural malformations present at birth and characterized by abnormal development of the heart and may be induced by environmental influence, altered gene function, or stochastic factors [1,2]. It is the most frequently diagnosed congenital disease affecting about 0.8–1.2% of live births worldwide [3]. Incidence and mortality from CHD vary globally. In the recent years, patterns, prevalence, and risk factors for CHD among newborns in Saudi Arabia have been studied. Interestingly, Kurdi et al. found in a three-year cohort case-control study that the prevalence rate for CHD was as high as 14.8 per 1000 births [4]. A plausible explanation for this comparable rate is consanguineous mating (inbreeding), a prominent phenomenon of the Arab countries that is widely accepted and practiced, particularly among first cousins. From a genetic standpoint, inbreeding has significant genetic implications for the offspring of inbred populations as it renders the genomes of the offspring autozygous due to identical chromosomal segments inherited from both parents. It is known form previous research that children born to closely related parents are at higher risk, approximately 2.0–2.5 times, of congenital malformations than the offspring of unrelated parents [5]. First-cousin marriage has been found to aggravate the estimation of malformation risk to 5–8% [6]. In Saudi Arabia, epidemiological reports have estimated that first-cousin marriages among families of children with CHD (41.6%) were significantly higher than the general population (28.4%) [7]. Therefore, the incidence of autosomal recessive congenital anomalies is expected to be higher in such highly inbred populations. Moreover, it has been shown that consanguinity increases the frequency of homozygosity for autosomal dominant traits, such as familial hypercholesterolemia, and possibly increases the prevalence of complex multifactorial conditions such as CHD [8].

Owing to the fact that Saudi Arabia has one of the highest consanguinity rates among Arab countries (>50%) besides its high fertility rate, it represents fertile ground for exploring the relevance of consanguinity to basic human genetics and applied clinical genetics [9]. Tracing back the biparental inherited recessive mutations of the same ancestral haplotype on which they reside has provided one of the most potent tools in interrogating the role of consanguinity in autosomal recessive disorders. Alkuraya has discussed a combination of techniques for mapping novel recessive disease genes. SNP-typing and Exome sequencing are powerful tools that can be used to trace the ancestral origin of homozygosity in autosomal recessive diseases [10]. Progressing further, another review by Alkuraya provided the impact of modern genomic tools, e.g., homozygosity mapping, next-generation sequencing, and molecular karyotyping, in mapping the consanguineous inheritance in Saudi populations, concluding with a remark addressing the issue of highly consanguineous populations. Long regions of homozygosity (ROH) leading to autozygosity are demonstrated to be universally found in human genomes, even among outbred individuals, according to genome-wide data [11]. In coming years, ROH analysis of short-read sequences of the entire genome or exome will be more familiar with reducing costs [12]. Here, we review the genomic studies performed in Saudi Arabia for the identification of genetic defects associated with elevated risk for CHD.

## 2. Consanguinity and CHD

The most detrimental consequences of consanguinity are frequent occurrences of autosomal recessive diseases in the offspring, leading to increased morbidity and mortality rates [13,14]. Encounters between people with two or more of these abnormal genes are frequently causing the presence of combination of pathogenic variant genes influencing the clinical presentation. Monies et al. reported the clinical exome sequencing (CES) on >2200 previously unpublished Saudi families as a first-tier test, highlighting 155 genes to be recessive, disease-related candidates in the Saudi population [9]. Autosomal-recessive variants account for 77.2% variants, 98.4% homozygous, and 41.3% founder variants [9]. The percentage of the genome rendered homozygous by consanguinity seems to be directly proportional to the degree of consanguinity [15]. In a population with a high consanguinity rate, the homozygous gene pool will inevitably include defective alleles. On the other hand, a similar mutation rate in consanguineous populations leads to increased allelic and even locus heterogeneity incidences. This is exemplified by Kamal et al. with a report of four novel mutations in the *ALMS1* gene among Saudi patients with very rare autosomal recessive Alstrom disease presenting allelic heterogeneity in this inbred population [16].

Chehab et al. performed one of the largest cohort studies to date (>1500 patients) to assess the connection between consanguinity and CHD incidence. This cohort was represented by 19% of first-degree cousins, 5.9% of second-degree cousins, and 2.1% of other parental consanguinity. Comparatively, the consanguineous contributions in the control group without CHD were: 14% (first degree cousins), 5.9% (second degree cousins), and 3.6% (other parental consanguinity), showing a significant difference between the two groups. However, consanguinity rates vary greatly between different types of CHD. The highest incidences of atrial septum defect (ASD), tetralogy of Fallot (TOF), and valvular aortic stenosis (AS) were among the defects with consanguineous parents, supporting the theory that these defects may be caused by recessive genes [17]. The prevalence of consanguinity among patients with CHD is also evidenced in many other studies [7,18,19]. Monies et al. [18] have undertaken the most extensive study on the spectrum of mutational genetic diseases in the Saudi population in the diagnostic setting. The random selection of tested families representing all regions of the Kingdom aided the inference of essential patterns of genetic diseases in a highly consanguineous Saudi population relevant to the broader community of diagnostic NGS labs worldwide. The first 1013 Saudi families’ first report was tested with seven gene panels and whole-exome sequencing (WES). A breakdown of the diagnostic yield by indication shows marked variability, with the highest yield being multiple congenital malformations in the prenatal setting followed by skeletal dysplasia. The duo testing of couples with deceased children was associated with a high diagnostic yield, reaching 83% when novel candidate genes are counted. Autosomal recessive pathogenic and likely pathogenic mutations accounted for 71% cases and 97% homozygous cases, consistent with the high rate of consanguinity (78% of 482 families that provided the consanguinity information) in this cohort. Of the recessive positive cases, 33% were due to founder mutations [18]. This report has highlighted the role of consanguinity in identifying important patterns of genetic diseases in the Saudi population that are relevant to the international community. The study has also highlighted the need for in-depth research to understand the Saudi population’s gene pool using NGS technologies. Parental consanguinity has been well proven to play a significant role in the prevalence of congenital heart disease in the Saudi population [19,20]. A study on a group of 1028 consecutive CHD patients identified through the Congenital Heart Disease Registry at King Faisal Specialist Hospital in the central region of Saudi Arabia, Riyadh, indicated that the proportion of first-cousin mating among CHD patients is significantly higher than that of first-cousin intermarriages reported in the general population. This study suggested that first-cousin consanguinity was significantly associated with ventricular septal defect (VSD), ASD, atrioventricular septa l defect (AVSD), pulmonary stenosis (PS), and pulmonary atresia (PA). Therefore, in a population with a high degree of inbreeding, consanguinity may exacerbate underlying genetic risk factors with prevalence of recessive component as causation of some cardiac defects [7]. Most recent cross-sectional studies found the prevalence of CHD in SA ranging between 2.1 and 10.7 per 1000 persons with VSD ranging from 29.5 to 39.5% followed by ASD (8.9% to 18.1%) and PS (6% to 12.4%). It was also stated that CHD occurrence in Saudi Arabia was significantly associated with Down’s syndrome, consanguinity, and maternal diabetes [20]. Comprehensive data on cohort studies conducted over the span of 25 years to identify the prevalence of CHD in the Saudi population indicated the significance of consanguinity as a risk factor, as summarized in Table 1.

Evidences of a genetic component in CHDs are familial recurrence and their association with inherited microdeletion syndromes [1]. Numerous CHD cases are known to be chromosomally abnormal, particularly those exhibiting syndromic characteristics such as multiple malformations of the organs, developmental delays, and growth abnormalities. Chromosomal aneuploidies are the first recognized genetic causes of CHD [33]. The condition affects nearly half of children born with trisomy 21 (one of 600) [34], 20–50% of babies born with Turner syndrome (one of 2500 female births) [35], and nearly all of those born with trisomy 13 and trisomy 18 [36]. A wide variety of cardiac malformations can occur with aneuploidy syndromes, but prototypic lesions are observed in trisomy 21 (AVSD) and Turner syndrome (CoA), while other lesions are underrepresented (e.g., TGA) [33]. Early findings from these genotype-phenotype observations suggested that malformations of the heart are not caused by a global change in genome content, but rather by altered doses of specific genes [33].

In Saudi Arabia, Al-Hassnan et al. [37] performed the first inclusive array CGH evaluation on Saudi cohort inflicted with CHD. They were able to identify cytogenetic imbalances among 17 selected CHD cases suffering from autism spectrum disorder, intellectual disability, developmental delay, language delay, or dysmorphic features of unknown origin. For instance, they detected a chromosomal deletion on chromosome 12p12.1 harboring *SOX5* in a patient born to consanguineous parents [37]. This finding attests to what has already been reported by several previous studies regarding the development of anomalies, such as ventricular septal defect, slight arrhythmia, Secundum atrial septal defect, and the atrioventricular canal, among CHD patients being linked to *SOX5* haploinsufficiency [38]. Through the same approach, Hussien et al. investigated genomic defects in a small cohort of children with syndromic CHD. They detected a high rate of cryptic chromosomal abnormalities or pathogenic CNVs clustered at certain chromosome loci shown as deletions/amplifications in 20% of the cases [39]. The findings of these cytogenetic studies highlighted the importance of array platforms in the genetic diagnosis of CHD in clinical care. However, it is essential to further analyze these clustered CNVs to identify candidate genes that may provide insight into the molecular mechanisms involved in CHD. In this regard, Alharbi et al. designed a comprehensive gene panel to analyze genetic defects associated with the CHD phenotype in Down’s syndrome patients both on chromosome 21 as well as autosomes other than chromosome 21. Based on the expression and function data, as well as pathogenicity of the variant of interest, they identified genetic defects in *GATA3*, *GUSB*, and *KCNH2* that are specific to DS patients with CHD [40]. Moreover, they found two splice variants in the FLNA gene in isolated DS patients and DS patients with CHD that they hypothesized might account for DS pathogenesis. They implicated the involvement of other genetic variations in abnormal cardiac development, including in *CEP290*, *ENG*, and *MEF2A* [40]. Furthermore, a very recent report by Dasouki et al. revealed a high yield of CNVs in a broad spectrum of CHDs, primarily in the chromosomal regions of chr 17q21.31, 16p11.2, 8p11.21, and 22q11.23 in a large cohort of Saudi patients. Subsequent functional and network analyses highlighted the involvement of several genes in CHD pathogenesis, including *NPHP1*, *PLCB1*, *KANSL1*, and *NR3C1* [41]. It is believed that the observed changes are enriched primarily in genes and pathways involved in cardiovascular function and development.

Numerous studies exploited the high incidence of monogenic disorders in Saudi Arabia to identify Mendelian phenocopies for complex disorders such as CHD. Shaheen et al. applied exome sequencing to three consanguineous families diagnosed with Truncus Arteriosus. In one of the patients suffering from syndromic CHD, a mutation was identified in the NRP1 gene which is predicted to result in premature truncation of the protein [42]. Moreover, Monies et al. explored the Saudi population genetic landscape for suspected Mendelian disorders using next generation sequencing methods on 1000 families spanning a wide range of suspected Mendelian phenotypes. Interestingly, recessive mutations accounted for 97% of the cases they solved. They noted the first instances of recessive inheritance of previously assumed strictly dominant disorders, including *MCTP2* involvement in CHD pathogenesis. Moreover, they identified novel recessive variants believed to be associated with syndromic CHD in the *PCNX* and *DMXL* genes, which have not previously been linked to human disease [18]. A couple of years later, Monies et al. (2019) reported additional recessive mutations that support the disease-causing candidacy of previously reported genes including *UNC5A* which were found to harbor two different recessive, likely pathogenic variants in a patient suffering from multiple congenital anomalies, including CHD with a failure to thrive phenotype [9].

Familial aggregation of heart anomalies has contributed significantly to our understanding of the molecular mechanisms behind many heart diseases. However, due to the complex nature of CHD pathogenesis, genetic heterogeneity, and multifactorial inheritance, it has proven difficult to identify disease-causing genes [32]. It was only through remarkable scientific advances, such as classical linkage analyses, positional cloning, and targeted sequencing of CHD-associated genes, that the rare Mendelian forms of CHD were discovered [43]. Many genes have been implicated for CHD phenotypes. However, evidence varies for each gene as a result of incomplete penetrance and variable expression of the cardiac phenotype, as seen in many familial cases of CHD. The first genes implicated in inherited non-syndromic CHD are transcription factors that regulate the expression of cardiac genes including *NKX2.5*, the *GATA* family, and T-box factors. Dominant mutations in these genes have been associated with inherited ASD, VSD, TOF, Ebstein’s anomaly, and cardiac conduction defects [44,45].

Exploring the genetic pool of the Saudi population has contributed significantly to the global effort of understanding Mendelian diseases, particularly in the area of autosomal recessive diseases, which account for the bulk of Mendelian disease burden. Sifrim et al. exome sequenced around 1900 patients with syndromic and non-syndromic CHD patients, including Saudi patients, and derived insightful conclusions. First, syndromic CHD is significantly enriched for de novo protein truncating variants (PTVs) but not inherited PTVs in known CHD-associated genes, consisted in consistent with several published reports. Second, conversely, non-syndromic CHD is significantly enriched for inherited PTVs in CHD-associated genes [46]. Mapping specific cardiac defects to regions of autozygosity in consanguineous families enabled Shaheen et al. to discover a homozygous PTV in PRKD1 in a Saudi family with non-syndromic CHD [42]. PRKD1 is listed in OMIM only as a CHD-associated gene in the context of de novo heterozygous mutation resulting in ectodermal dysplasia with CHD. However, recent studies [9,47] not only provided confirmation that PRKD1 is indeed associated with recessive, non-syndromic truncus arteriosus, but also expanded the CHD phenotype associated with this gene. There are several additional novel genes that have been identified in the Saudi population associated to isolated forms of CHD, including *PRDM1* and *ADAMTS19* [42,48].

Despite the increasing evidence that some types of CHD are inherited recessively, recessive inheritance of CHD remains poorly understood. Using NGS technologies to identify many CHD recessive genes in consanguineous populations has facilitated our understanding, but a combination of other molecular approaches, including mutagenesis screening in model organisms, may speed up our findings.

## 3. Current Perspectives and Future Directions

Understanding the global, regional, and national distribution of CHD is essential for developing various strategies for prevention. There has been a noticeable increase in the prevalence over the past decade, which has been attributed to the improvement in CHD screening and pediatric care. However, in most cases, the primary cause is unknown hindering accurate predictions of familial recurrence risk and prenatal diagnosis. The effort of large-scale collaborative sequencing projects and family-based studies offer key evidence in support of a genetic component to CHD. Yet, the clinical implementation of these approaches has been hampered by low yield expectations and inconsistent reporting practices of clinical variants. The proper implementation of genetic testing in daily clinical practice requires clear reporting from laboratories, an accurate interpretation by clinicians, and consistent communication with patients regarding the implications of their findings. The development of a classification system for variants with specific recommendations for action for each classification would assist in achieving this objective. Clinical interpretation of sequence variants is not restricted to testing for CHD susceptibility. Genetic testing has a variety of applications, including risk prediction, carrier testing, and reproductive decision-making, which may impact how variants are classified.

In light of the Saudi culture, the high consanguinity and birth rates, and the increased burden of genetic diseases, the challenge for health-care strategists in the country is to find solutions that are easily implemented and sustainable over time. Understanding the socio-cultural characteristics of Saudi Arabia is essential. Consanguinity is on the decline globally according to [49,50]. However, the rate in Saudi Arabia has not shown an appreciable decline in more than a decade [51,52], indicating a pressing need for an exhaustive plan to increase public awareness and improve access to genetic services for preventative purposes.

## Figures and Tables

**Table 1:** Spectrum of epidemiological and consanguinity-associated congenital heart disease (CHD) studies in Saudi Arabia.

Study Reference	City	Study Setting	Data Collection Year	CHD Sample Size	Findings
Prevalence and Relative Frequency Studies					
[21]	AlQassim	Hospital	1988–1991	320	Relative frequency of VSD (38.5%) was higher ASD (11.5%), Pulmonary Stenosis (PS) (9%), PDA (8%) and AVSD (5%)
[22]	AlMadina	Hospital	1992–1995	1209	VSD (29.7%), ASD (26%), PS (16.1%) and PDA (13.2%)
[23]	Asir	Hospital	1994–1996	335	VSD (32.5%), PDA (15.8%); ASD (10.4%); PS (10.1%); AVSD (3.6%) and mitral valve prolapse (3.6%); CoA (3.3%); obstructive aortic valve lesions (2.7%); TOF (4.5%); common ventricle (2.7%); PA with VSD (1.8%); D-transposition of the great arteries (1.5%); Ebstein anomaly (1.5%) and PA (1.2%).
[24]	Nation-wide	National registry	1998–2002	5865	The Southwestern region exhibits the highest burden of CHD. AlBaha with a prevalence estimate of 748/100,000.
[25]	Hofuf	Hospital	1997–2000	740	VSD was the most common defect (39.5%), followed by ASD (11.5%), PS (8.9%), PDA (8.6%), AVSD (3.5%), TOF (4.2%), CoA (2.7%), AS (3.5%)
[26]	Nation-wide	Household	2004–2005	95	The highest prevalence in the central region (27/10000). Northern and Eastern had a prevalence of (25/10,000) while the Southwestern region had a prevalence of 21/10,000. VSD was the most common defect (10/10,000).
[27]	AlMadina	Hospital	2007–2008	4348	CHD represents 34.4% of all cardiac problems. VSD represented 34.5% of all CHD diagnoses, followed by ASD (8.9%), PS (7.9%), PDA (6%), AVSD (3.8%), TOF (3%), AS (3.5%), CoA (2.8%), TGA (3.5%), and others (26%)
[28]	Albaha	Hospital	2005–2010	2610	VSD (29.6%), PDA (9.5%), ASD (9.3%), PS (7.9%), AVSD (6.0%), TOF (4.7%), COA (3.4%), AS (3.0%), and TGA (1.9%)
[4]	Riyadh	Hospital	2010–2013	1179 CA cases	The birth prevalence of CA was 412/10 000 births, driven mainly by CHD (148/10,000). Isolated CHD found in 62.5%, distributed as VSD (28%), ASD (25.3%), PA and PS (6.8%) and severe CHD (20.4%)
[29]	Albaha	Hospital	2016–2017	2961	CHD was diagnosed in 49 patients of the positive test group, (1.7%) distributed as 5 (0.2%) patients with VSD, and 44 (1.5%) patients with large symptomatic PDA.
[30]	AlMadina	Hospital	2017–2019	1127	The acyanotic CHDs were the predominant lesions, accounting for 84.8% of all cases, while the cyanotic types accounted for 13%. PDA VSD, ASD, CoA and AVSD represented 27.9%, 24.8%, 18.9%, 6.4%, and 4.4% of the total cases, respectively. TOF (8.7%), followed by TGA (1.7%) and TA (1.1%), were the most common cyanotic CHDs.
**Consanguinity-Associated Studies**					
[7]	Riyadh	CHD Registry	1998	949	There was a significantly higher incidence of CHD among first-cousin marriages (41.6%) in comparison to the general population (28.4%).
[31]	Riyadh	CHD Registry	1998	891	It was found that consanguinity was significantly higher in the sample (40.4%) than in the general population (28.4%). Some forms of CHD are significantly associated with consanguinity, such as VSD, ASD, AVSD, PA, and PS, but not TOF, TA, AS, COA, or PDA.
[32]	Dhahran	Hospital	1996–2000	37 families	There were 23 consanguineous marriages (62%) in these families. Dilated cardiomyopathy was more common in consanguineous marriages; 26 cases vs. 2 in non-consanguineous marriages
[19]	Nation-wide	Household	2004–2005	11,554	CHD was the only disease associated with first cousin consanguinity in 56% of respondents.

Abbreviations: CHD, Congenital heart disease; VSD, ventricle septum defect; ASD, atrial septum defect; PS, pulmonary stenosis; PDA, patent ductus arteriosus; PA, pulmonary atresia; TOF, tetralogy of Fallot; AVSD, atrioventricular septum defect; COA, coarctation of the aorta; TA, tricuspid atresia; AS, aortic stenosis; TGA, transposition of the great arteritis; CA, congenital anomaly.3. The Genetic Landscape of Non-Syndromic CHD.

## Data Availability

Not applicable.
